# Vestibular paroxysmia: clinical characteristics and long-term course

**DOI:** 10.1007/s00415-022-11151-6

**Published:** 2022-05-20

**Authors:** Karoline Steinmetz, Sandra Becker-Bense, Ralf Strobl, Eva Grill, Klaus Seelos, Doreen Huppert

**Affiliations:** 1 German Center for Vertigo and Balance Disorders, Ludwig-Maximilians-Universität München, Marchioninistrasse 15, 81377 Munich, Germany; 2 Institute for Medical Information Processing Biometry and Epidemiology (IBE), Ludwig-Maximilians-Universität München, Munich, Germany; 3 Department of Neuroradiology, Ludwig-Maximilians-Universität München, Munich, Germany

**Keywords:** Vestibular paroxysmia, Neurovascular compression, Symptomatology, Vestibular testing, Long-term course

## Abstract

In 2016, the Bárány Society defined new diagnostic criteria for the neurovascular compression syndrome of the eighth nerve, called “vestibular paroxysmia” (VP), differentiating between definite (dVP) and probable (pVP) forms. The aim of this study was (1) to describe clinical symptoms and laboratory findings in a well-diagnosed large patient cohort according to those criteria, and (2) to evaluate the long-term course over years in dVP. We identified 146 patients (73 dVP, 73 pVP) from our tertiary dizziness center registry. Data of structured history-taking, clinical neurological, neuro-ophthalmological/-otological examinations as well as MRI imaging were extracted for analyses. Overall, attack frequency ranged between 5 and 30 attacks per day; spinning vertigo was the most frequent type. In two-thirds of patients, attacks occurred spontaneously; in one-quarter, they were triggered by head movements. The majority (approximately 70%) reported no accompanying symptoms; in those with symptoms, mild unilateral cochlear symptoms prevailed. One-third of patients initially showed hyperventilation-induced nystagmus without specific direction, and a deviation of the subjective visual vertical between 3° and 6°. Complete loss of peripheral vestibular function was never evident. dVP and pVP significantly differed concerning the vertigo type, e.g., spinning vertigo was more frequent in dVP. Fortunately, three-quarters of dVP patients remained attack-free during follow-up (mean 4.8 years, standardized questionnaire), more than half of them even without any medication. Patients with ongoing attacks showed significantly higher attack frequency at baseline, but reported persistent frequency reduction. Overall, the long-term prognosis of VP appears favorable, not necessarily requiring ongoing treatment.

**Supplementary Information:**

The online version contains supplementary material available at 10.1007/s00415-022-11151-6.

## Introduction

In 1975, Jannetta and colleagues were the first who described a neurovascular compression syndrome of the eighth cranial nerve [1], which they named “disabling positional vertigo”[2]. The term “vestibular paroxysmia” (VP) was introduced in 1994 by Brandt and Dieterich, who also proposed the first diagnostic criteria [3]. Recently, new diagnostic criteria for VP were defined by the Classification Committee of The International Bárány Society [4] differentiating between definite and probable forms (Supplementary Table 1). The core symptoms are frequently recurring, short attacks of spinning or non-spinning vertigo lasting for seconds to minutes. VP is a rare disease, but it is regularly seen in specialized dizziness centers [5, 6].

The underlying mechanism is assumed to be a pathologic vessel–nerve contact, mostly by the anterior inferior cerebellar artery (AICA), inducing demyelinisation with succeeding hyperexcitability enabling ephaptic depolarization [3, 7, 8]. The intra-cisternal part, where the nerve is myelinated by oligodendroglia, is assumed to be the most vulnerable segment for this syndrome [9]. The currently favored hypothesis is that VP is an excitatory rather than a hypofunctional vestibular phenomenon [10]. The demyelinisation in the root-entry zone is usually not visible in standard MR imaging [1, 3, 10, 11]. However, in symptomatic patients, a potential vessel–nerve contact is found in > 95% in 1.5 T magnetic resonance imaging reflecting high sensitivity [8, 12, 13]. Since approximately 30% of healthy adults also show such a vessel–nerve contact [8, 12, 13], its specificity is low and therefore imaging is not part of the current diagnostic criteria. A therapeutic trial of low-dose sodium channel blockers, e.g., carbamacepine/oxcarbacepine, is often effective, and is required for the diagnosis of definitive forms of VP [4]. However, the long-term course of VP is not entirely clear. The only available study in 32 VP patients reported a significant initial decrease in attack frequency, intensity, and duration during treatment [12], reflecting a more principal proof of effectiveness. Furthermore, there are only a few observatory studies available concerning the symptomatology and laboratory tests that mostly refer to former diagnostic criteria not differentiating between probable and definite forms of VP and/or to small patient cohorts [8, 10, 12, 14].

Therefore, the aim of this patient cohort study was 2-fold:

1. to describe the clinical symptoms and laboratory vestibular findings in a large well-diagnosed patient cohort from a tertiary dizziness center according to the current diagnostic criteria of definite VP and probable VP, and
2. to evaluate the long-term clinical course over years.

## Materials and methods

### Patients and study design

We screened the patient registry (DizzyReg) of our tertiary dizziness center (German Center for Vertigo and Balance Disorders, DSGZ, Munich, Germany) for all adults with suspected diagnosis of VP. DizzyReg aims to collect all clinical data in a standardized way to create a comprehensive clinical database of patient characteristics, symptoms, diagnostic procedures, diagnosis, therapy, and reported outcomes [15]. Inclusion criteria into the registry are age 18 years and above, signed informed consent, and sufficient knowledge of German. VP patients were categorized as probable VP (pVP) or definite VP (dVP) according to the current diagnostic criteria of the Classification Committee of the International Bárány Society [4]. Patients with relevant neurological, otological, neurodegenerative (e.g., encephalomyelitis, Parkinson’s disease), or any other additional vestibular diseases were excluded. Ethical approval of the Ludwig-Maximilians Universität München ethics board was obtained in accordance with the ethical standards laid down in the 1964 Declaration of Helsinki and its later amendments (protocol no. 414-15). In retrospect, 146 patients fulfilled the current diagnostic criteria for VP: 73 (50%) for pVP and 73 (50%) for dVP. Overall, the mean age was 49.76 ± 12.91 years (70 females, 76 males), equally distributed between dVP and pVP (for details see Table 1).

**Table 1:** Patient characteristics and symptoms

Patient characteristics	Definite VP (dVP)		Probable VP (pVP)		dVP vs. pVP
	Mean	SD	Mean	SD	*p* value
Age	50.34	±12.57	49.18	±13.3	0.5866
Gender	m/f (%)		m/f (%)		0.136
	59/41		45/55		
Symptoms	*n*	%	*n*	%	
Type of vertigo					**0.0469**
Spinning	44/73	60	31/73	42	
Non-spinning	29/73	40	42/73	58	
Frequency of attacks					0.2562
5–9 × daily	24/67	36	34/73	47	
10–29 × daily	35/67	52	28/73	38	
≥ 30 × daily	8/67	12	11/73	15	
Spontaneous attacks	50/73	68	49/73	67	0.9412
Triggered attacks	23/73	31	24/73	33	
Head movements	19/73	26	19/73	26	
Physical activity	4/73	5	5/73	7	
No accompanying symptoms	53/73	72	51/73	70	1
Accompanying symptoms	20/73	28	22/73	30	
Cochlear symptoms	10/73	14	14/73	19	0.5029
Nausea	7/73	10	5/73	7	0.7632
Oscillopsia	3/73	4	1/73	1	0.6122
Head pressure	0/73	0	2/73	3	0.4765

Values in bold type are statistically significant values

### Clinical examination and instrument-based vestibular testing

All patients underwent structured history-taking and standardized physical examination at the German Center for Vertigo and Balance Disorders, including neurological and neuro-ophthalmological examinations (fundus photography, head-shaking test, subjective visual vertical (SVV), and hyperventilation test (clinically with Frenzel glasses and with the scanning laser ophthalmoscope). A mean SVV deviation of more than ± 2.5° from the true vertical was considered as pathological. Standardized laboratory neuro-otological testing comprised bithermal water calorics testing of the horizontal canal and horizontal video head impulse test (vHIT; EyeSeeCamHIT^®^ system, Interacoustics, Middelfart, Denmark^®^) [16]. Values above 25% side asymmetry during caloric testing were considered pathological [17]. A vHIT gain of less than 0.7 was considered pathological [18], as was a vHIT gain asymmetry index of more than 10%.

### Follow-up in definite VP

All patients with dVP presented at least twice in our center. To evaluate the long-term course, including symptoms and medical history, a standardized telephone interview was performed in 2021 in 38/73 patients with dVP (Supplementary Figure 1) by an experienced neurootologist. In addition, 5 patients completed the questionnaire independently in writing. We finally collected completed questionnaires from 43 patients, corresponding to a response rate of 59%. The remaining patients could not be tracked, mostly because of incorrect telephone contact or address data due to the long follow-up period.

### cMRI evaluation in definite VP

A neuroradiologist (Kl.S.) re-evaluated the MRI scans, primarily on the basis of T2-weighted 3D sequences (CISS, FIESTA, SPACE) with slice thicknesses of 0.5 to 0.8 mm. In approximately 50%, an additional non-contrast-enhanced pure flow-based 3D TOF MRA with slice thicknesses of 0.6 to 1.2 mm was available. Contrast agent was given in only three cases. In cases, in which the original images were missing, we included written documentation from external neuro-/radiologists, if available.

### Statistical analyses

After data collection, all data were irreversibly anonymized for data analyses; the statistic program R (Version 3.6.1) was used for all analyses [19]. For data description, we used mean values and standard deviation for continuous variables and absolute and relative frequencies for categorical variables. We performed the *t* test to calculate for differences in continuous variables and Pearson’s chi-squared test for categorical variables. Statistical differences between participants with dVP vs. pVP, as well as between attack-free vs. persons with ongoing attacks were calculated. Logistic regression was applied to analyze factors associated with being attack-free. Model fit was tested by the Hosmer–Lemeshow statistic, with a non-significant result (*p* > 0.05) indicating adequate fit [20]. Age, gender, number of attacks at baseline, caloric findings, and results of the SVV were included in the model. Statistical significance was set at a two-tailed 5% level.

## Results

### Symptoms and laboratory examinations in dVP

At first presentation, none of the later diagnosed dVP patients were on specific medical treatment. Spinning vertigo was reported by 60% and non-spinning vertigo by 40%. The majority (52%) initially had 10–29 attacks per day. History-taking revealed that the attacks occurred spontaneously in 68%, were triggered by head movements in 26%, and by physical activity in 5%. Most patients (70%) reported no accompanying symptoms. In those who did report accompanying symptoms, they were most frequently mild cochlear ones (14%), such as unilateral tinnitus, hearing impairment and/or ear pressure, followed by nausea in 10% and only occasionally oscillopsia (4%) (Table 1).

SVV measurements were normal in the majority of patients (82%). In 18% (*n* = 13) mild binocular SVV deviation (mean 3.92°) as a sign of acute vestibular tone imbalance even in the symptom-free interval was found without side preference (rightwards: 5/13, mean 3.75°, range + 3° to + 4.5°; leftwards: 8/13, mean − 4.03°, range − 3° to − 6°). A differentiation between ipsi- and contraversive tilting was not useful, since the causative side could not be consistently identified. Hyperventilation-induced nystagmus was detected in 42% (*n *= 19/46) of patients, equally often in vertical (*n* = 8/19, downbeat > upbeat) and horizontal direction (*n* = 11/19). Caloric testing showed mild side asymmetry (25–35%) in only 10 patients (14%). The results of the video head impulse test were mostly unremarkable; only 6 (11%) showed a slightly reduced gain unilaterally (0.6–0.7). No patient showed complete peripheral functional loss. Table 2 summarizes all examination results.

**Table 2:** Results of vestibular testing

Clinical examination	Definite VP (dVP)		Probable VP (pVP)		dVP vs. pVP
	*n*	%	*n*	%	*p* value
Subjective visual vertical					0.1823
Rightward deviation	5/72	7	3/73	4	
Leftward deviation	8/72	11	3/73	4	
Hyperventilation ind. nystagmus	19/46	42	19/50	38	0.1647
Upbeating	5/46	11	4/50	8	
Downbeating	3/46	7	10/50	20	
Rightward	5/46	11	3/50	6	
Leftward	6/46	13	2/50	4	
Caloric irrigation					0.576
Reduction right	7/68	10	4/71	6	
Reduction left	3/68	4	4/71	6	
Video-head-impulse test					0.4109
Reduction right	5/53	9	3/56	5	
Reduction left	1/53	2	0/56	0	

Neuroradiological reevaluation of the original MRI data confirmed in 19/21 patients a neuro-vascular contact (NVC) between the N. vestibulocochlearis and a blood vessel, whereas in two cases, no NVC was evident. In the majority of cases (95%; *n* = 18), the compressing vessel was the AICA, only once (5%) the PICA. Bilateral contact was seen in 12 out of 19 cases, unilateral contact in 7. The mean distance from the NVC to the brainstem was 7.4 ± 2.7 mm (2.0–13.5 mm) on the right and 6.8 ± 2.9 mm (1.0–13.0 mm) on the left (for details see Table 3). In further 25 patients, a present vessel–nerve contact was earlier documented by an externally neuro-/radiologist, in 9/25 on the basis of CISS-/FIESTA-sequence, and in 16 only by standard MRI. NVC was unilateral in 22 and bilateral in three cases.

**Table 3:** Reevaluation of original MRI scans in definite vestibular paroxysmia

Case	Neurovascular contact right			Neurovascular contact left		
	Vessel	Localization NVC	Distance to brainstem (mm)	Vessel	Localization NVC	Distance to brainstem (mm)
1	AICA	cisternal	4.7	–	–	–
2	AICA	cisternal	7	AICA	cisternal	8
3	AICA	cisternal	7	AICA	intrameatal	8
4	AICA	intrameatal	8	AICA	cisternal	4
5	AICA	intrameatal	13	AICA	cisternal	8
6	AICA	cisternal	8.5	–	–	–
7	PICA	cisternal	5	AICA	cisternal	7
8	AICA	intrameatal	13.5	AICA	cisternal	5
9	AICA	cisternal	9	–	–	–
10	AICA	intrameatal	12	–	–	–
11	AICA	intrameatal	7	AICA	cisternal	5
12	AICA	cisternal	8	AICA	intrameatal	13
13	AICA	cisternal	4	–	–	–
14	AICA	cisternal	7	AICA	extrameatal	7
15	AICA	cisternal	7	AICA	cisternal	7
16	AICA	cisternal	2	–	–	–
17	AICA	cisternal	7	–	–	–
18	AICA	cisternal	7.5	AICA	cisternal	1
19	AICA	intrameatal	8	AICA	cisternal	9
			Mean + SD			Mean + SD
			7.4 ± 2.7			6.8± 2.9

### Symptoms and laboratory examinations in pVP

In pVP, 42% reported spinning and 58% non-spinning vertigo, 47% of them fewer than 10 attacks per day and 38% 10–29 attacks per day. The attacks occurred spontaneously in 67% and were triggered by head movements in 26%, and by physical activity in 7%. The majority (68%) showed no accompanying symptoms. If accompanying symptoms were present, there were mainly mild unilateral cochlear symptoms (19%), and rarely nausea, head pressure or oscillopsia (Table 1).

Binocular SVV measurement showed a slight deviation in only 8% (mean 3.94°), equally distributed to the right (mean 4.3°, range + 2.7° to + 5.6°) and to the left (mean 3.58°, range − 3.25° to − 3.5°). Hyperventilation-induced nystagmus was found in 38% (*n* = 19/50) of patients, 14/19 vertical (downbeat > upbeat) and 5/19 in horizontal direction. Caloric irrigation was asymmetric in 12% (25–35% side difference) and the video head impulse test in 5% (10–20% side difference) (Table 3). Even in this subgroup, no complete peripheral vestibular dysfunction was detected.

Vessel–nerve contacts were reported by externally assessed cMRI in 23 patients, unilaterally in 18 patients (3 using CISS sequence) and bilaterally in 5 (2 using CISS sequence).

### Comparative analyses between dVP and pVP

The mean age of patients with definite and pVP was similar (50.3 vs. 49.2 years; *p* = 0.5866); dVP tended to prefer male (♂ 59%: ♀ 41%), whereas pVP female gender (♂ 47%:♀ 53%), both without statistical significance (Table 1).

Patients with dVP initially reported spinning vertigo significantly more often (60% to 42%; *p* = 0.0469). There was no statistical significant difference in terms of frequency of attacks, although frequency in dVP mostly ranged between 10–29 times daily (52%) and in pVP in the other frequency ranges (predominantly 5–9 times daily: 47%). In both groups, the majority of attacks occurred spontaneously (dVP 68% vs. pVP 67%), and were triggered similarly often either by head movements (26%) or physical activity (dVP 5% vs. pVP 7%). The majority of patients presented only with monosymptomatic vestibular complaints (dVP 72% vs. pVP 70%). If accompanying symptoms were present, mild unilateral cochlear ones were most common without significant difference (dVP 19% vs. pVP 14%). All other accompanying symptoms showed a similar distribution (see Table 1).

There were no significant differences for SVV deviation (dVP 18% vs. pVP 8%), hyperventilation-induced nystagmus (42% vs. 38%), partial caloric deficits (14% vs.12%), and gains in the video head impulse test (11% vs. 3%) (Table 2).

In summary, the only parameter that significantly differed between the two groups was the type of vertigo at symptom onset, which was significantly more often of spinning type in dVP.

### Medical treatment and long-term progress in dVP

After the first outpatient visit, all patients responded to the therapy with carbamazepine (200–1000 mg daily) or oxcarbazepine (300–900mg daily), and so could be classified as dVP at follow-up visit. The vast majority took a dosage of 400–600 mg carbamazepine (200 mg *n* = 2, 400 mg *n* = 34, 600 mg *n* = 16, 800 mg *n* = 8, 900 mg *n* = 1, 1000 mg *n* = 2) and 300–450 mg oxcarbazepine (300 mg *n* = 4, 450 mg *n* = 4, 900 mg *n* = 2). Thirteen (18%) of the 73 dVP patients switched the initial medication in the course due to treatment side effects (fatigue, dizziness, liver enzyme increase, and occasionally depressive mood or decrease in libido) to gabapentine (*n* = 7), lacosamide (*n* = 5), or phenytoin (*n* = 1).

In a total of 43/73 (response rate 59%) dVP patients, it was possible to assess the long-term progress over a period up to 11.8 years by the standardized interview. The mean follow-up time was 4.8 years (median: 4.0 years).

**Fig. 1:**
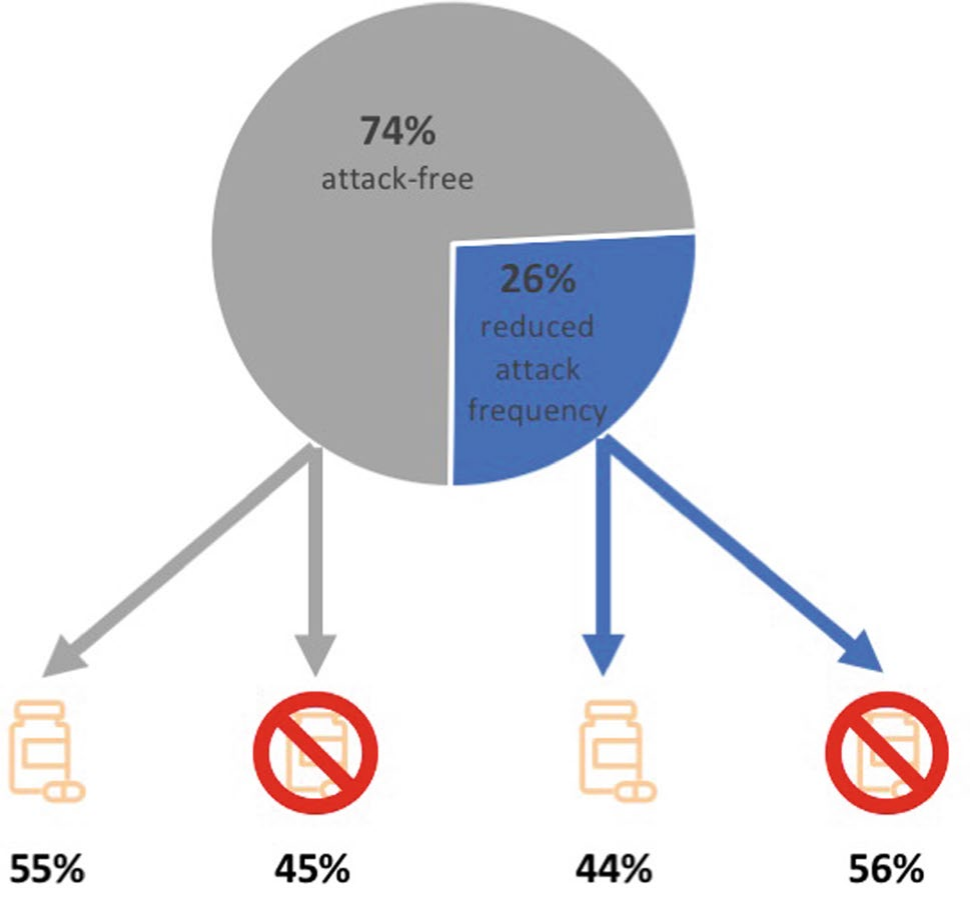
Synopsis of long-term follow-up in definite vestibular paroxysmia

The majority of patients became totally attack-free (74%) in the long-term course, 56% of them without any medication and 44% with continued medication. However, in the 26% (*n* = 11) with ongoing attacks, the frequency remained significantly reduced in the long-term course, in 45% with, and in 55% even without medication (Figure 1). All patients were treated for a mean period of 6.5 months. In the attack-free subgroup, discontinuation was mainly due to freedom from symptoms, except in two cases with side effects.

To identify potential predictors for freedom from symptoms in the long-term course, we compared patient characteristics, initial clinical features, and examination results between the attack-free and still symptomatic patient groups. Patients who remained symptomatic, initially showed a significantly higher frequency of vertigo attacks than patients who became attack-free (attack-free*:* 43%: 5–9, 43%: 10–29, 13%: > 30 daily attacks vs. ongoing attacks: 0%: 5–9, 82%: 10–29, 18%: > 30 daily attacks; *p* = 0.0285). All other parameters did not significantly differ between the two groups: age (51.31 years ± 14.64 vs. 48.45 years ± 8.91; *p* = 0.5437), gender (♂:♀= 66:34% vs. ♂:♀ = 45:55%; *p* = 0.4106), type of vertigo (*p* = 0.4106), triggers (*p* = 0.6525), accompanying symptoms, SVV deviation (*p* = 0.3964), hyperventilation-induced nystagmus (*p* = 0.6413), caloric deficits (*p* = 0.6292), pathological gains in vHIT (*p* = 0.8347). Furthermore, the symptoms lasted for a similarly long period in the attack-free and the still symptomatic patient group (19 vs. 22 months) before initiation of medical treatment. However, the logistic regression revealed that besides the higher attack frequency at onset of more than 10 attacks daily (OR = 1.38; *p* = 0.0453), also female sex (OR = 1.32; *p* = 0.0415) was associated with persistence of symptoms in the long-term course. The 10% of patients without detectable vessel–nerve contact in MRI showed symptom refractoriness to a similar proportion than those with proven vessel–nerve contact. Furthermore, a clear correlation between the initial CBZ dosage on attack frequency reduction as well as in the long-term course was not detected.

## Discussion

In our patient cohort study, we identified 146 patients with VP according to the newest diagnostic criteria of the Bárány Society [4]. This well-examined cohort served as a good basis for symptom and clinical characterization as well as for statistical comparison. Additionally, in 43 out of 73 dVP patients a long-term follow-up could be assessed. The key findings of our study were as follows:

1. Mean age at symptom onset was about 50 years without gender preference.
2. The most frequent type of vertigo was spinning in dVP (60%) and non-spinning in pVP (58%), which was the only significant difference in clinical characteristics between the two groups. Attack frequency typically ranged between 5 and 29 attacks per day; frequencies above 30 times per day were rare. In two-thirds of patients, the attacks occurred spontaneously, in a quarter, they were triggered by head movements. The majority of patients reported no accompanying symptoms (Table 1).
3. Hyperventilation-induced nystagmus was evident in about one third of patients, SVV-deviation in 18% of dVP. Complete peripheral vestibular deficits (bithermal calorics and vHIT) was not found at all. There were no significant differences in the laboratory examinations between dVP and pVP (Table 2).
4. In 90% of all available cMRIs (in dVP), a unilateral or bilateral NVC could be found mostly cisternal (distance to the brainstem 7.1 mm), typically by the AICA (95%) and followed by PICA (5%) (Table 3).
5. In dVP, three-quarters of patients remained attack-free in the long-term course, more than half of them even without any medication, in the quarter of patients with ongoing attacks, the frequency was significantly reduced. In the latter, the initial attack frequency was significantly higher (≥ 10 attacks daily).

Vestibular paroxysmia is a relatively “young” disease with its first systematic description by Brandt and Dieterich in 1994 [3]. There are no data available on lifetime prevalence in this rare entity, but in specialized tertiary dizziness centers, it is regularly diagnosed [5, 6]. Over the years, the diagnostic criteria have been repeatedly amended, most recently in 2017 by the Bàràny Society [4]. Currently, for dVP at least 10 short, stereotyped attacks of rotational or non-rotational vertigo below 1 minute duration as well as a response to medical treatment (CBZ/OXC) are required (Supplementary Table 1). For the category of pVP, only five attacks for less than 5 minutes are necessary. Previously, a selection of associated symptoms (e.g., unsteadiness of stance and gait, unilateral cochlear symptoms) or additional criteria (e.g., NVC on MRI with CISS sequence, hyperventilation-induced nystagmus, or progressive vestibular deficits) were taken into account [3, 7, 8, 12], but did not find their way into the current classification.

Overall, studies on the symptomatology, laboratory examinations, and long-term follow-up referring to the current criteria are rare. The only available study refers to a smaller cohort of 22 patients not differentiating between dVP and pVP [14]. The two most relevant and carefully conducted earlier patient cohort studies by Hüfner et al. (2012) and Best et al. (2009) on 32 and 20 patients respectively, refer to former diagnostic criteria and/or had a different study focus. Thus, our study aimed to investigate the symptomatology and examination results in a large patient referring to the current criteria separately for the subgroups of dVP and pVP. It was our particular intention to also evaluate the long-term course over years after the diagnosis of dVP.

Our data confirmed the earlier described findings of a mean age at symptom onset around of 50 years with equal gender distribution in dVP as well as pVP, despite limitations in comparability [8, 12, 14] (Table 1).

In our study, the most frequent type of vertigo in dVP was spinning (60%) compared to pVP with 42%, which was the only significantly different clinical parameter between the two subgroups. Thus, the high occurrence of rotational vertigo of around 70% in the above-mentioned studies was confirmed for dVP, but not for pVP. This might be due to relatively weak inclusion criteria for pVP, requiring only 5 short attacks of vertigo-like sensations, thereby risking including non-vestibular attacks, e.g., due to circulatory dysregulations. This result appears to be logical, since in VP the peripheral vestibular system is involved, typically reflected by rotatory vertigo.

Most probably due to the fact that specific triggers and accompanying symptoms are not mandatory in the current diagnostic criteria, spontaneous occurrence was two to three times more frequent and accompanying symptoms, especially cochlear ones, less frequent in our cohort. Overall, about 70% of patients reported no accompanying symptoms at all and if they were present, they occurred equally often in dVP and pVP, so they did not help to differentiate between the two entities.

Hyperventilation-induced nystagmus is suggested to reflect hyperexcitability of the compressed nerve, but is also found in other peripheral vestibular diseases, e.g., vestibular schwannoma and unilateral vestibulopathia [21, 22], as well as in vestibular migraine, cerebellar diseases [23], and even in healthy adults (up to 12%) [24, 25]. In our patient cohort, hyperventilation-induced nystagmus was found in about 40% of dVP as well as pVP patients, which is less frequent compared to earlier descriptions of up to 70% [12].

All available studies that investigated SVV deviation showed increased rates of pathological tilting in smaller cohorts (22% [12] to 40% [8]). This is approximately in line with the rate of 18% slight SVV deviations in our study only in dVP, whereas it was low in pVP (8%). The persisting mild tone imbalance might explain ongoing unspecific impairment in the attack-free interval, e.g., unsteadiness of gait or drowsiness. In contrast to hyperventilation-induced nystagmus, SVV deviation as a sign of an acute vestibular tone imbalance is not at all evident in healthy persons, and thus can serve as a more reliable indicator. In contrast, mild peripheral dysfunction (calorics, vHIT) can also be explained by preceding diseases, and does not imperatively reflect acute pathology.

The vestibulocochlear nerve is especially susceptible to compression due to its much longer central myelin portion compared to other cranial nerves [26]. It has a long cisternal segment, which extends from the brainstem to the internal auditory canal and has a length of 14.2–19.2 mm [27]. In line with earlier descriptions, the offending vessel in our study was the AICA in the vast majority of available imaging data. The distance of the neurovascular contact, which varied from 0 to 10.2 mm in previous studies [6], ranged in our study at comparable values between 1.0 and 13.5 mm.

In a high-resolution 7 Tesla cMRI study of definite VP patients, no structural lesion of the nerve induced by the vessel–nerve contact could be confirmed [10], which supported the hypothesis that VP is rather an excitatory than a lesion-induced phenomenon. One can speculate about the source of VP-like symptoms in the 10% of our patients without MRI proven vessel–nerve contact that other excitatory phenomena of the eights nerve, e.g., spontaneous electrical membrane discharges, or central paroxysms caused minute brainstem lesions might mimic VP. In the latter, a comparable response to sodium channel blockers is to be expected; even in our subgroup without proven vessel–nerve contact, there was no increased rate of refractory patients. However, the fact that direct vessel–nerve contact is not verifiable in all patients is also found in other vascular nerve compression syndromes, such as hemispasmus facialis or trigeminal neuralgia [28, 29].

Overall, the long-term course in dVP was found to be favorable with three-quarters of patients remaining attack-free over years, surprisingly half of them even without any medication, and in the patients with ongoing attacks with a significant reduction of attack frequency. This finding further supports the hypothesis that VP is more an excitatory rather than a hypofunctional phenomenon caused by structural damage [6]. The sometimes monophasic occurrence or cessation of symptoms is in line to other vascular nerve compression syndromes as, for example, in glossopharyngeal and trigeminal neuralgia [30, 31]. Recovery from symptoms could, for example, be due to declining contact by vessel elongation with consecutive remyelinisation. Also in children, a favorable course of VP was reported and explained by remission due to processes of maturation [32, 33].

Differences between the two groups of patients (still attacks vs. attack-free) were only observed regarding the frequency of attacks at the beginning (≥ 10 attacks in logistic regression analyses) and female gender. However, these two parameters do not allow any prognostic predictions about the individual course of the disease.

The general difficulty in VP is that it is a purely clinical diagnosis that cannot be clearly proven by any objective parameter. Sodium channel blockers have further effects on serotonin systems, and are mood-stabilizing too [34]. The currently required symptomatology, especially in pVP of only five non-spinning vertigo attacks for less than 5 minutes, can be caused by a variety of complaints including psychosomatic diseases, circulatory dysregulations, or atrial fibrillations, all of which can manifest without additional specific symptoms beside the sensation of vertigo and dizziness. Since medication seems to contribute significantly to the initial improvement and is required to diagnose dVP forms, a treatment attempt appears reasonable, especially in patients with frequent short attacks of rotatory vertigo. However, due to our positive long-term data, a careful dose reduction or even slow drug withdrawal after a minimum period of about 6 months appears justifiable.

The main limitation of the current study is its potential patient selection bias due to the referral of patients to a specialized tertiary center, which does not allow a transfer to the general population. However, arguably, this limitation is offset by the rigorousness of data collection in our prospective patient registry. Further prospective studies on prognosis and treatment, especially placebo-controlled double-blind trials considering also the long-term course, should be conducted.

## Supplementary Information

Below is the link to the electronic supplementary material.

Supplementary Figure 1: Follow-up questionnaire in definite vestibular paroxysmia (DOCX 13 KB)

Supplementary Table 1: Diagnostic criteria for vestibular paroxysmia Diagnostic criteria of definite and probable vestibular paroxysmia according to the consensus document of the Bárány Society 2016 [4]. All criteria listed in the table have to be fulfilled in (DOCX 13 KB)

## Data Availability

All data are available from the corresponding author.
