# Supplementary material for: Vestibular paroxysmia: clinical characteristics and long-term course
Source: J Neurol. 2022 May 20;269(12):6237–45. doi: 10.1007/s00415-022-11151-6 (PMC9618515; doi:10.1007/s00415-022-11151-6)
Supplement: Supplementary file 1 — Supplementary Figure 1: Follow-up questionnaire in definite vestibular paroxysmia (DOCX 13 KB) [file 415_2022_11151_MOESM1_ESM.docx]

**Supplementary Figure 1: Follow-up questionnaire in definite vestibular paroxysmia**

| Name: Date of birth:  1. Do you currently still have vertigo attacks?  Yes: No:  If no:  Since when?  How long did the symptoms last?  If yes:  Duration?  Frequency?  Type of vertigo?  Trigger?  Accompanying symptoms?  Changed compared to before?  Other medication in the coure of time?  2. Do you still take your medication?  Yes: No:  If yes:  Which one?  What dosage?  If no:  How long have you been taking the medication?  When was treatment ended?  Why was treatment ended (e.g. due to side effects or absence of symptoms)?    3. Did you have to change the medication due to side effects or insufficient effect during the course of time?  Yes: No:  If yes:  Due to side effects:  Due to insufficient effect:  Change from (medication named) : 🡪 to  4. Please send us your cMRI (MRI of the skull) for a new neuroradiological evaluation to  the following address:  Deutsches Schwindel- und Gleichgewichtszentrum  z.H. Dr. Karoline Steinmetz  Marchioninistr. 15  81377 München.  We will of course send it back to you. |
| --- |
