# Supplementary material for: Vestibular paroxysmia: clinical characteristics and long-term course
Source: J Neurol. 2022 May 20;269(12):6237–45. doi: 10.1007/s00415-022-11151-6 (PMC9618515; doi:10.1007/s00415-022-11151-6)
Supplement: Supplementary file 2 — Supplementary Table 1: Diagnostic criteria for vestibular paroxysmia Diagnostic criteria of definite and probable vestibular paroxysmia according to the consensus document of the Bárány Society 2016 [4]. All criteria listed in the table have to be fulfilled in (DOCX 13 KB) [file 415_2022_11151_MOESM2_ESM.docx]

**Supplementary Table 1**

| Definite vestibular paroxysmia | Probable vestibular paroxysmia |
| --- | --- |
| At least 10 attacks of spontaneous spinning or non-spinning vertigo | At least 5 attacks of spinning or non-spinning vertigo |
| Duration less than 1 minute | Duration less than 5 minutes |
| Stereotyped phenomenology in a particular patient | Spontaneous occurence or provoked by certain head-movements |
| Response to a treatment with carbamazepine/ oxcarbazepine | Stereotyped phenomenology in a particular patient |
| Not better accounted for by another diagnosis | Not better accounted for by another diagnosis |
